# Identification of the Proliferation/Differentiation Switch in the Cellular Network of Multicellular Organisms

**DOI:** 10.1371/journal.pcbi.0020145

**Published:** 2006-11-24

**Authors:** Kai Xia, Huiling Xue, Dong Dong, Shanshan Zhu, Jiamu Wang, Qingpeng Zhang, Lei Hou, Hua Chen, Ran Tao, Zheng Huang, Zheng Fu, Ye-Guang Chen, Jing-Dong J Han

**Affiliations:** 1 The Chinese Academy of Sciences Key Laboratory of Developmental Biology, Center for Molecular Systems Biology, Institute of Genetics and Developmental Biology, Chinese Academy of Sciences, Beijing, People's Republic of China; 2 The State Key Laboratory of Biomembrane and Membrane Biotechnology, Department of Biological Sciences and Biotechnology, Tsinghua University, Beijing, People's Republic of China; Weill Medical College of Cornell University, United States of America

## Abstract

The protein–protein interaction networks, or interactome networks, have been shown to have dynamic modular structures, yet the functional connections between and among the modules are less well understood. Here, using a new pipeline to integrate the interactome and the transcriptome, we identified a pair of transcriptionally anticorrelated modules, each consisting of hundreds of genes in multicellular interactome networks across different individuals and populations. The two modules are associated with cellular proliferation and differentiation, respectively. The proliferation module is conserved among eukaryotic organisms, whereas the differentiation module is specific to multicellular organisms. Upon differentiation of various tissues and cell lines from different organisms, the expression of the proliferation module is more uniformly suppressed, while the differentiation module is upregulated in a tissue- and species-specific manner. Our results indicate that even at the tissue and organism levels, proliferation and differentiation modules may correspond to two alternative states of the molecular network and may reflect a universal symbiotic relationship in a multicellular organism. Our analyses further predict that the proteins mediating the interactions between these modules may serve as modulators at the proliferation/differentiation switch.

## Synopsis

Coordination of proliferation and differentiation is a fundamental process of multicellular organisms. Although at the cellular level proliferation and differentiation seem to correspond to different cellular states that can sometimes be seen separated by the proliferation/differentiation temporal switch, it is unclear whether such switch-like property exists at the tissue or organism level or whether it exists in postmitotic tissues in adult animals. Through integrating protein–protein interaction networks with gene expression profiles, Xia, Xue, Dong, Zhu, and colleagues found that a switch temporally separating proliferation- and differentiation-associated modules can also be detected in the adult human brain and the adult whole fruit fly. The expressions of the two modules are well coordinated at the system level. The evolutionary origins of the proliferation and differentiation modules further implicate a symbiotic relationship between the two modules. Network topologies and gene annotations support a regulatory role of the protein–protein interaction interface between the two modules.

## Introduction

How cells coordinate proliferation and differentiation has been one of the most important questions in developmental biology, cell biology, and cancer biology. The idea that growth and proliferation are poorly compatible with differentiation has wide currency, and explicit proliferation/differentiation switches have been demonstrated for many different cell types [1–4], but no general mechanism has been apparent. Due to the multifactor nature of this coordination process and the recent advances in gene networks, Waddington's theory of development as a canalization of the epigenetic landscape shaped by gene networks [5] has gained more popularity. As predicted by this theory, a breakthrough may be achieved through a systems approach. Recent production of various “-omics” data has probed the gene networks from various aspects. Integrating the static interactome together with the expression and phenotypic profiles during a certain biological process can frequently reveal the dynamics of the gene network [6,7].

The protein–protein interaction networks (PPI, or interactome networks) have been shown to have dynamic modular structures [7,8], yet the functional connections between and among the modules are less well-understood. Through examining the dynamics of the interactome network, we found that two major network modules, the “P” (for proliferation) and “D” (for differentiation) modules are anticorrelated transcriptionally over adulthood in both the human brain and the fruit fly. These modules are enriched in proliferation and differentiation genes, respectively, and display alternatively lower and higher expressions at the cellular proliferation/differentiation switch. Most P module genes are conserved between higher organisms and unicellular organisms such as yeast, but most D module genes are absent from unicellular organisms. Thus, these modules may correspond to alternative cellular states characteristic of higher organisms.

## Results

### Transcriptionally Anti-Correlated Modules in the Interactome Network

To investigate the dynamic features of the human interactome network through changes in gene expression, we used as surrogates the expression profiles on 30 postmortem human brains from subjects ranging from 26 to 106 years old. This dataset was originally generated to examine the age-related changes in gene expression and the biological functions related to aging [9]. In this study, we only focus on the gene expression patterns across different individuals and try to dissect the modular structure of the interactome network by similar or opposite expression profiles between a pair of genes.

As both transcriptional correlation and anti-correlation between a pair of genes are biologically relevant under specific conditions [10,11], we focused on the subnetwork that consists of only interactions between gene pairs that are transcriptionally correlated and anti-correlated (abbreviated as correlated and anti-correlated interactions, respectively) to examine the dynamic modular structure of the interactome. Such networks will be referred to as the NP network, where NP stands for negative and positive correlations. The expression correlations and anti-correlations between a pair of genes are commonly measured by a correlation coefficient. The Pearson correlation coefficient (PCC) is known to focus on the “shape” of changes rather on than the intensity or amplitude of signals, and hence does not have bias for strong signals and has been shown to be best suited for oligo arrays [12,13]. It has a value of 1 for perfect correlation or −1 for perfect anti-correlation.

**Figure 1:**
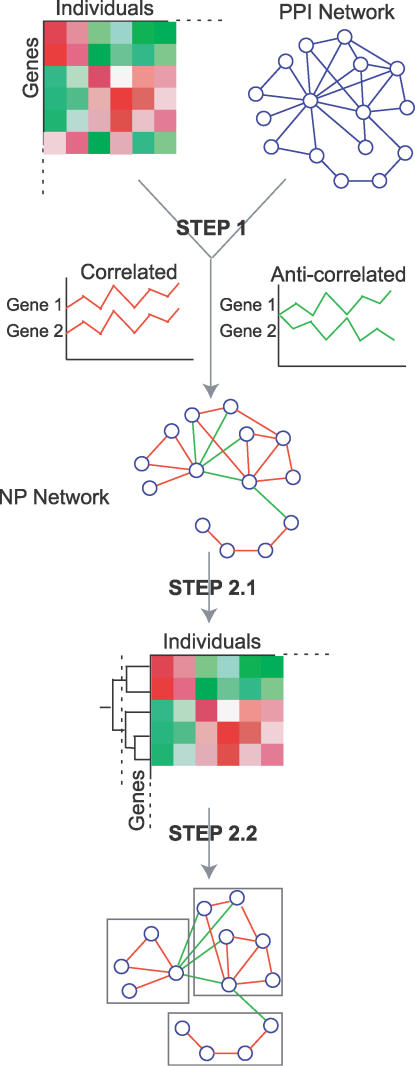
The Analysis Pipeline Used to Reveal the Anti-Correlated Modules The analysis includes two major steps: step 1, calculating pairwise PCC for the transcriptional profiles of each pair of genes engaged in a PPI to extract the NP network from the PPI network; and step 2, applying hierarchical clustering to the genes in the NP network (step 2.1), then manually dissecting the largest anti-correlated clusters, or scanning from the top of the hierarchical tree for clusters that have <1% intracluster anti-correlated interactions (step 2.2) to approximate the goal of obtaining modules within which the expression profiles of genes are similar and correlated interactions are maximally enclosed, and in between which anti-correlated interactions are optimally distributed. A more detailed textual flowchart is available in Figure S1.

Our analysis pipeline includes the following steps (Figures 1 and S1): (1) obtain all the PPIs between genes that have similar expression profiles or opposite expression profiles determined by PCCs as correlated or anti-correlated interactions to arrive at the NP network; (2) identify network modules so that the expression profiles of genes within a module are similar, correlated interactions are maximally enclosed within a module, and anti-correlated interactions are optimally distributed between modules. The second step is approximated by first applying hierarchical clustering to the genes in the NP network, then manually dissecting the largest anti-correlated clusters or automatically scanning from the top of the hierarchical tree for clusters that have <1% intracluster anti-correlated interactions and finding the largest anti-correlated clusters with an average expression of PCC < −0.7.

Extracted from PPIs in the Human Protein Reference Database (HPRD) [14], the NP network across the human brain frontal cortex expression profiles [9] comprises 1,055 correlated and 395 anti-correlated interactions among 1,260 genes/proteins. We used PCC values of 0.4 and −0.4 as cutoffs for positive and negative correlations, respectively. These cutoffs have been established in previous studies. However, as described later, the identities of the clusters are not dependent on PCC cutoffs.

**Figure 2:**
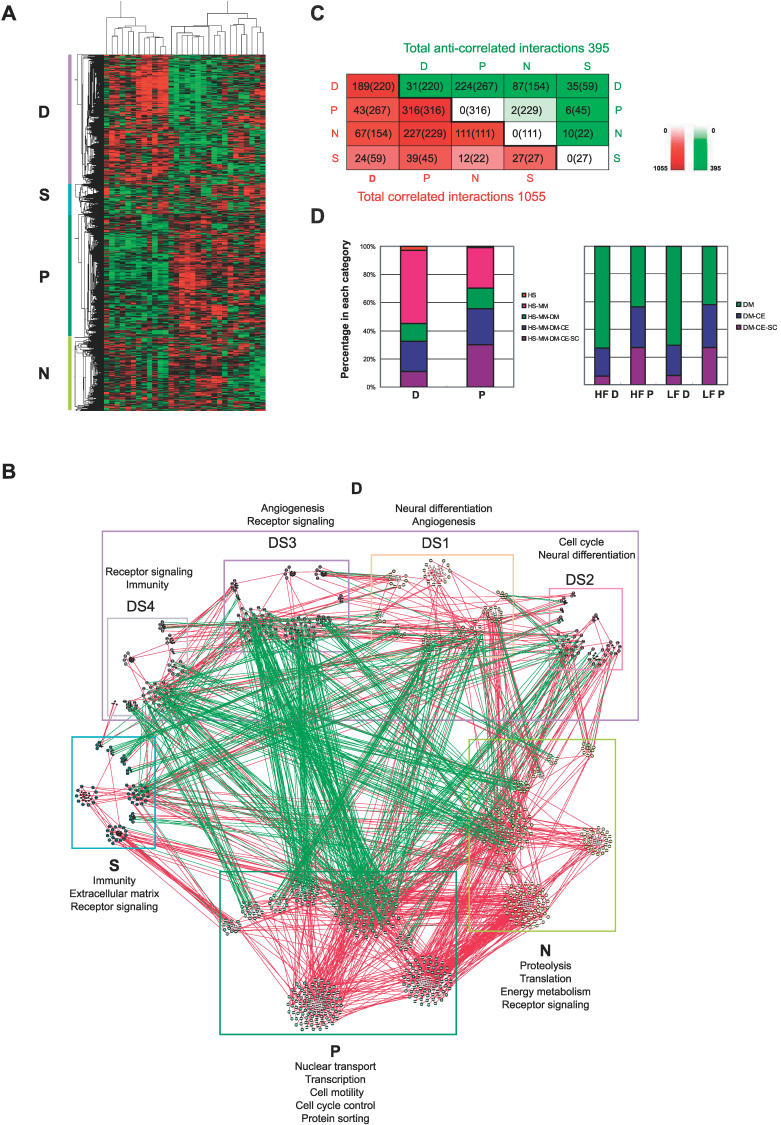
The Transcriptionally Anti-Correlated Modules in the NP Network (A) Two of the four major gene clusters, the D and P clusters, among genes in the NP network are anti-correlated. Human brain samples were clustered on the horizontal dimension according to their expression similarity. Genes were clustered on the vertical dimension. When the D gene cluster is upregulated, the P gene cluster is downregulated, and vice versa. (B) The NP network is reorganized to display the extensive anti-correlated interactions from D towards both the P and N modules, which are themselves linked by only correlated interactions, and the few anti-correlated interactions within the D module that separate the module into subgroups DS1 to DS4. Red edges represent correlated interactions; green edges, anti-correlated interactions. Nodes in the largest component of the network are shown in larger size. The most enriched biological functions of each cluster are listed beside the module labels. (C) The number of anti-correlated and correlated interactions within and between the D, P, N, and S modules. Red denotes correlated interactions; green, anti-correlated interactions. Color intensity indicates the proportion of interactions among the total correlated or anti-correlated interactions. (D) Evolutionary origins of P and D modules. Both the human and fruit fly P modules are more conserved from the single-cellular organism yeast to the multicellular organisms, whereas the D modules are more specific for multicellular organisms, with the human D module especially specific for human and mouse. Human genes in each module are divided into five different categories: those that are conserved among yeast, worm, fly, mouse, and human (purple); those that are conserved among worm, fly, mouse, and human (blue); and so on (green and pink); those that are only found in human are red. Fly genes are divided into those that are conserved among yeast, worm, and fly; among worm and fly; and those that are fly-specific. SC, CE, DM, RN, MM, and HS stand for yeast *Saccharomyces cerevisiae,* fly *Drosophila melanogaster,* worm *Caenorhabditis elegans,* rat *Rattus norvegicus,* mouse *Mus musculus,* and human *Homo sapiens,* respectively. HF and LF stand for “high food” and “low food”, respectively.

Using the hierarchical clustering algorithm implemented by Cluster [15,16] and visualizing the clusters with Tree View [15,17], we found that most of the anti-correlated interactions in the NP network bridge between two anti-correlated expression clusters among the genes (nodes) within the NP network. This is apparent if the samples are clustered on the other dimension (Figure 2A). We named the two anti-correlated clusters “P” and “D” based on their associations with proliferation and differentiation functions, respectively (see below). Another large cluster that is largely correlated with P, but not obviously anti-correlated with D is named the “N” cluster for “not determined” function. The smallest cluster is named the “S” cluster for “small” (Figure 2A). There are 457, 435, 260, and 108 nodes, and 220, 316, 111, and 27 interactions within the D, P, N, and S clusters, respectively (Figure 2B and 2C). Genes in each module are listed in Table S1.

To examine the biological functions of each module, we first searched for overrepresented Gene Ontology (GO) categories among the genes within a module. To fully illustrate the preference of certain biological processes in one module versus others, we further grouped the related GO terms into a few broad categories, and performed a more comprehensive keyword search for genes potentially sharing the same process but not annotated by GO; these are listed at the end within each category in Table 1. The genes associated with the overrepresented GO terms and those found by the keyword search were also listed. According to Table 1, the D cluster is enriched in circulation/angiogenesis, apoptosis machinery, and ion and neurotransmitter channels, which are hallmarks of neural differentiation, cell cycle regulators, cell surface receptors, and steroid receptors. The P cluster is enriched in transcription, nuclear and intracellular transport, cell cycle, and cell motility genes. These enriched GO terms suggest that the P and D modules might be associated with cell proliferation and differentiation processes, respectively. The N cluster is enriched in genes involved in proteolysis, translation activity, intracellular transport, and energy metabolism. The S cluster is related to immunity (Table 1 and Figure 2B). A subset of the D genes (253 genes from D and one gene from N), the “SD” cluster, also anti-correlates with a subset of the N genes (250 genes from N and 40 genes from D), the “SN” cluster, across different subgroups of human subjects (Figure S2A and S2B).

**Table 1:**
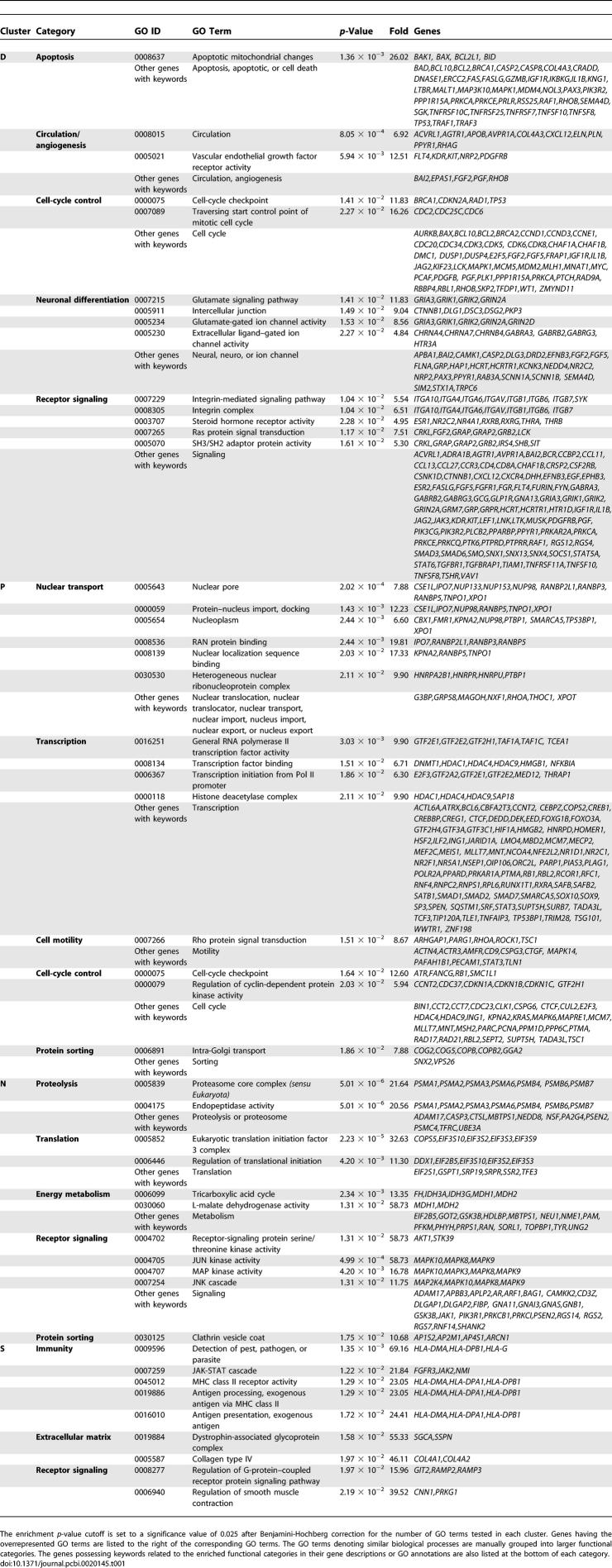
GO Terms Overrepresented in Each of the Gene Expression Clusters

The anti-correlated expressions of the D and P clusters are even more evident when the average gene expression levels of these clusters in each sample are compared across different human subjects, with a PCC reaching −0.867; that is, in almost each case when the expression of D is up, that of P is down, and vice versa.

Since genes and the functional relationships among the genes in a coexpressed cluster are frequently called a coexpressed module in a network [18], we will refer to these clusters as modules in the context of a network. The D module can be divided into four smaller submodules, DS1 to DS4, by reclustering only the D genes so that no anti-correlated interactions are within any submodules (Figure 2B). These submodules are, however, connected by more correlated than anti-correlated interactions (Figure S2C).

### The P and D Modules Reflect the Dynamics of the Cellular Network

If the P–D partition is a feature of the dynamic transcriptional regulation in the adult brain, we would expect that the partition should not be dependent on whether or not the PPI network is integrated and should also be independent of the PCC cutoffs used to extract the NP network.

To test whether the physiological transcriptome is necessary for the P–D partition, or if network topology alone may give rise to such partitions, we permuted the expression values of each gene among different samples in the HPRD network, calculated the PCC of each HPRD interaction, and identified coexpressed modules using the automated pipeline (Figure 1). Among 100 such permutations, none of them gives rise to a pair of anti-correlated modules of more than 100 nodes per module (empirical *p* < 0.01; Control 1 in Table 2). Permuting gene expression intensities within each sample (Control 2 in Table 2), or permuting PCC values among different PPIs (Control 3 in Table 2) also renders the anti-correlated modules undetectable or barely detectable (*p* < 0.01 and *p* = 0.08, respectively). These randomization controls verify that the P–D partition is a true nature of the expression patterns, and cannot be derived by randomized expression patterns or pairwise relationship of expression profiles. As the networks in these controls have exactly the same network topology as the HPRD network, they also demonstrate that HPRD topology alone is not sufficient to give rise to the P–D partition. In other words, the P–D partition is not an artifact of network topology. In fact, it does not depend on any particular network at all.

**Table 2:**
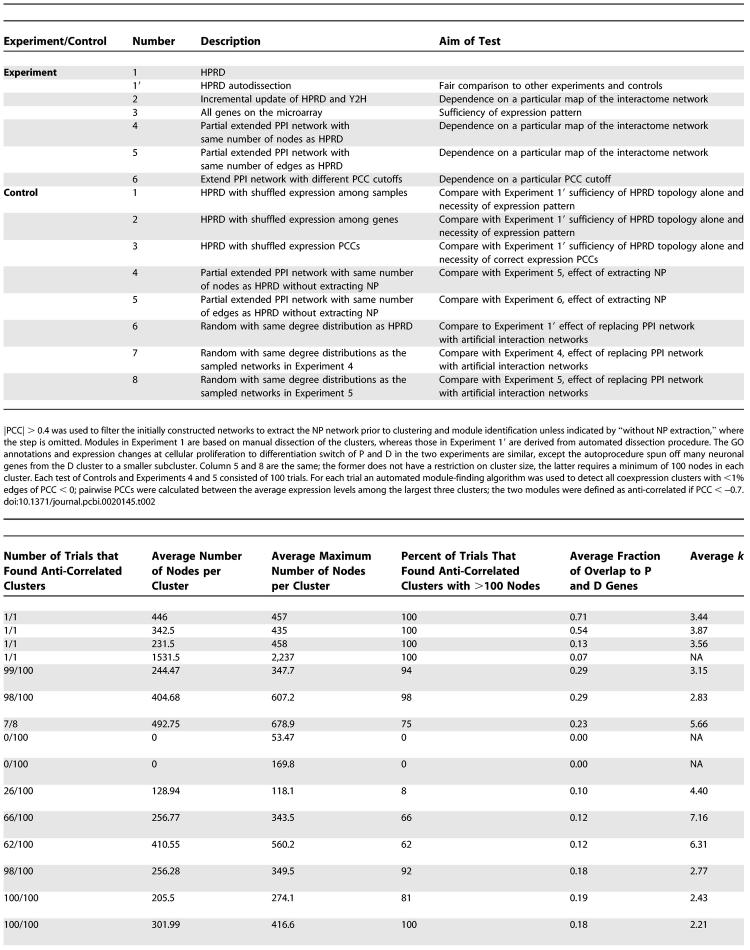
Probability of Obtaining P–D Partition

We created a nonoverlapping PPI dataset to the original early version of HPRD interactions that consists of the incrementally updated interactions to HPRD since the earlier version (the interactions added to HPRD between November 22, 2004, and September 13, 2005) and two recently generated Yeast Two-Hybrid (Y2H) datasets [19,20]. With this different PPI dataset, similar P, D, and N modules can be extracted from the NP network (Experiment 2 in Table 2 and Figure S3) that indicate the presence of P, D, and N modules is not a bias introduced by the original collection of HPRD interactions. Loosely aggregated coexpression clusters can be also derived from all genes on the microarray to significantly overlap with P (783 nodes) and D (1,276 nodes) modules (Experiment 3 in Table 2). The P–D partitions can actually be detected in almost any network of the same number of nodes (Experiment 4 in Table 2) or the same number of edges (Experiment 5 in Table 2) as the HPRD network but randomly sampled from an extended PPI network. The extended PPI network contains the updated HPRD plus two yeast two-hybrid interactome maps [19,20] and covers 7,568 proteins, 3,973 of which have expression profiles on the Affymetrix U95 array.

The P–D partition also does not depend on any particular PCC cutoff used to extract the NP network. In the extended interactome, using the automated module dissection pipeline at various PCC cutoffs or even without a PCC cutoff (|PCC| > 0), we could identify large clusters of genes that share significant overlaps with the P, D, and N modules. Although they correspond to smaller and smaller fractions of the total genes available on the microarray when the |PCC| cutoff increases, the fraction of genes corresponding to P and D modules maximizes around |PCC| cutoffs of 0.45 and 0.5 (Text S1 and Figure S4). Furthermore, relationships among the P, D, and N clusters did not change; only the number of genes and interactions varied to some extent without altering most of the enriched functions in each clusters (unpublished data). Therefore, although P and D modules are identified as predominate modules in the NP network of |PCC| > 0.4, the two modules reflect the dynamics of the whole cellular network, which is not limited to those covered by the NP network.

### Stable Module Detection by Integrating the Interactome with the Transcriptome

As demonstrated above, the presence of P, D, and N modules are not dependent on a particular PPI network being examined or a particular PCC cutoff. Why then do we need to integrate the PPI network and extract the NP network? The answer lies in the difference in the stability of detecting these network modules.

Unlike the gene clusters in the NP network, where 71% of the genes fall into P and D clusters, when all the genes in the full HPRD network are clustered, a pair of loosely aggregating anti-correlated gene clusters covers only 24% of the HPRD genes (Experiment 3 in Table 2, Text S1, and Figure S4). Similarly, the anti-correlation between the SD and SN was not visually clear when all the genes in the NP network were clustered (Figure 2A). Only after we examined the distribution of the anti-correlated interactions, of which a large number between the D and the N modules are evident (see below), we decided to further cluster only the genes in the D and N clusters. Then, an obvious anti-correlation between the SD and SN clusters became visible (compare Figure 2A and Figure S2A). These suggest that by concentrating on only the correlated and anti-correlated interactions, we enriched for the genes of the P, D, and N clusters.

To more rigorously test the stability of finding the P–D partition, we compared the chance of finding anti-correlated network modules with 100 or more nodes in each module and their overlap to the P and D modules with or without extracting NP networks prior to clustering the genes in the network. The chance of detecting anti-correlated modules is 99%, or 98% when an NP network with a |PCC| > 0.4 was extracted from a randomly chosen partial PPI network with the same number of nodes or edges as the early version of the HPRD (Experiments 4 and 5 in Table 2). By omitting the step of extracting the NP network, the chances are reduced to 66% and 62%, respectively. In addition, the fraction of P and D module genes among all the input genes also reduces from an average of 29% of total input genes to 12% (compare Experiments 4 and 5 with Controls 4 and 5, respectively, in Table 2).

Choosing a different PCC cutoff also reduces the chance to 75% (Experiment 6 in Table 2, Text S1, and Figure S4), with the fraction of genes identified as P and D module genes reaching maximal levels at |PCC| > 0.45 or |PCC| > 0.5 (Text S1 and Figure S4). If the PPI network is replaced by a randomly generated network with the same degree of distribution as HPRD or a sampled PPI network, using |PCC| > 0.4 as a cutoff to extracting the NP network, although the chance of identifying anti-correlated modules are still high, these anti-correlated modules are of smaller average sizes and display low overlap (18%–19%) with P and D modules (compare Controls 6–8 with Experiments 1, 4, and 5, respectively, in Table 2). These reductions in the probability of finding anti-correlated modules and further reductions in the identification of P and D modules among the input genes (or all modules) point to a role of using the appropriate PCC cutoff and integrating true PPIs on the stability of P and D module identification. However, the contribution of integrating the PPI network is not limited to the module identification, but more importantly is linked to the identification of the large PPI interface between the P and D modules that potentially coordinate the cellular proliferation and differentiation processes (see below).

These controls demonstrate that integrating the interactome, extracting the NP network, and applying an appropriate PCC cutoff ensured a high probability of stably detecting the P and D modules and improved their homogeneity, probably by filtering out most gene pairs that function in irrelevant tissue or cell types or under irrelevant physiological conditions.

### Conservation of P–D Anti-Correlation in Other Species

The P–D partitions and their transcriptional anti-correlation can also be seen in the fruit fly. We used the adult whole-fly expression profiles to probe the dynamic gene relationships in the network. In the original publication [21], the expression profiles were used to study the effect of diet restriction on aging, and consisted of two sets of profiles: one for flies fed with a large amount of food, and one for those fed with a small amount of food, called “high-food” and “low-food” conditions, respectively. Here, we used these profiles to extract the network modules based on anti-correlated and correlated interactions across different fly populations using the automated analysis pipeline described in Figure 1. The modules derived under high-food and low-food conditions are more than 50% identical. While the composition of the P module is largely conserved between the human brain and the fly, that of the D module is quite different between the two species. In particular, the apoptosis pathways are only enriched in the human brain D module. The enriched differentiation markers in D modules are also different; in the human brain, there are the neuronal markers; in the fly, there are genes involved in eye development (unpublished data), which is consistent with their tissue- and organism-specific requirements for differentiation.

We examined the percentages of human gene orthologs that can be found in yeast, worm, fly, and mouse. We found that 60% of D genes are specific to mouse and human and only 8% have yeast origin, whereas 35% of P genes have yeast homologs, and less than 30% are mammalian-specific (Figure 2D). Similar evolutionary patterns can be seen for fly P and D modules (Figure 2D).

The above observations indicate that the P module is more conserved from the single-cellular organism yeast to the multicellular organisms *C. elegans, Drosophila,* mouse, and human, while the D is multicellular-specific and is subjected to species-specific and probably also tissue-specific modifications.

The conservation of the P–D partition, their relationship, and the similar evolutionary profiles between fly and human also indicate that these observations cannot be due to sample variations introduced by sample preparations or other technical factors, but instead reflect true biological features of the gene networks of different multicellular organisms.

### The P/D Temporal Switch Corresponds to the Proliferation and Differentiation Switches

A switch between differentiation and proliferation has been demonstrated in myoblast C2C12 cells [1]. Inhibition of the P–D interface protein HDAC4 has been shown to promote differentiation and inhibit proliferation, whereas inhibition to another interface protein, SRF, does the reverse [1]. Both the HDAC4 and SRF proteins are downregulated upon differentiation with a concurrent increase in differentiation markers and their antagonizing microRNAs [1]. The levels of α_5_β_1_ integrin bound to fibronectin have also been shown to control the switching between proliferation and differentiation of C2C12 cells [4]. A proliferation/differentiation switch has also been observed in neural progenitor cells, and PI3K, cyclic AMP, raf, and MAPK pathways, which are all present at the P–D protein interaction interface, have all been implicated in regulating the switch [2,3]. These findings and many others collectively point to the existence of the switch between proliferation and differentiation at the cellular level.

**Figure 3:**
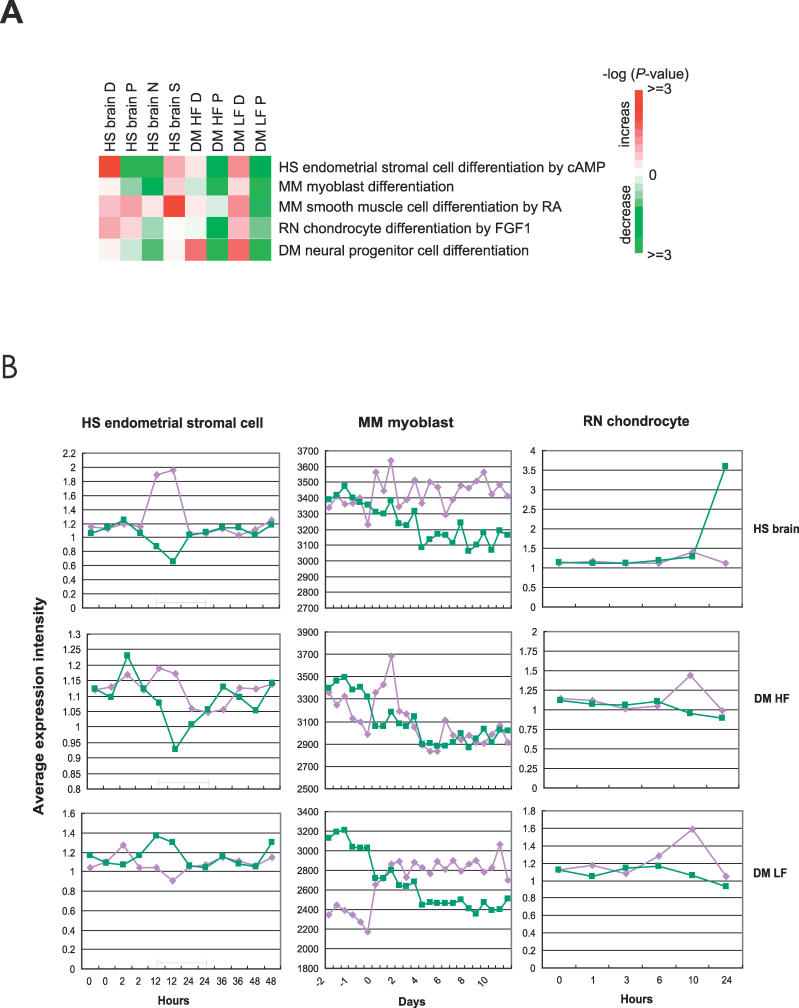
P and D Modules Correspond to the Proliferation/Differentiation Switch (A) The suppression of P and induction of D expression upon cellular differentiation. The expression levels of genes in the human brain P, D, N, and S modules and fly P and D modules under high-food (HF) or low-food (LF) conditions (listed in column headers) are compared between the undifferentiated and differentiated samples (listed in row headers) by paired Student *t*-test. Red and green colors indicate an increase and a decrease in the differentiated samples, respectively. The color intensity represents the –log (*p-*value) of the Student *t* test between the undifferentiated and differentiated samples. See text and Table S2 for details about the cell lines and differentiation conditions. (B) The average expression level of P and D genes during the time course of the cell differentiation process of the human endometrial stroma cell (left column plots), the mouse myoblast (middle column plots), and the rat chondrocyte (right column plots). The plots in the top row are the average expression of human brain P and D genes (left column) or their mouse (middle column) or rat (right column) homologs upon induction of differentiation; plots in the middle and bottom rows are those of the human, mouse, and rat homologs of the fly genes in P and D modules derived under high- and low-food conditions, respectively. The expression levels of P and D modules are indicated by the green and lavender lines, respectively. The suppression of P and induction of D can occur during a short window. The proliferation/differentiation time window has been marked by a red line for human endometrial stroma cell differentiation (left column plots) as annotated by the original paper [33] where the experiments are published. The other experiments presented in panel A but not here include only single timepoints.

If the P and D modules are indeed associated with the proliferation and differentiation processes as suggested by the enriched GO annotations, we expect they might correspond to the cellular proliferation/differentiation switch in the tissue and organism we examined. Indeed, we found a decrease of P expression and an increase of D expression when fly, rat, mouse, or human cells of various cell types are switched from the proliferation to the differentiation state upon induction by various external stimuli. In this analysis, we used previously published data on human endometrial stromal cell differentiation induced by cyclic AMP, mouse C2C12 myoblast differentiation upon shifting to differentiation medium, mouse smooth muscle cell differentiation induced by retinoic acid, the inhibition of proliferation and induction of differentiation by the FGF of rat chondrocytes, and fruit fly neural progenitor cell differentiation (detailed sample information is available in Table S2). Consistent with the conservativeness of the P module, P is more uniformly suppressed upon differentiation of various different tissues in various different organisms. For example, the expression of fly P genes, especially of those derived under diet restrictions or low-food conditions, is suppressed in all cell types (Figure 3A, and middle and bottom rows in Figure 3B). In contrast, the expression of the human brain D is strongly induced during human endometrial stromal cell differentiation, and less so during mouse and fly cell differentiation; the expression of fly D genes is only most strongly induced in fly cells, but less so in cells of other organisms (Figure 3). Furthermore, detailed time courses of the proliferation/differentiation switch revealed that the P/D transition occurs only at the exact short window of the switch and are not observed before or after the switch (human endometrial stromal cell in Figure 3B), which accounts for some weak signals when the expression levels of all timepoints before or after the switch are averaged (Figure 3A). In addition to the association to proliferation and differentiation processes suggested by the overrepresented functional annotations in the P and D modules and transcriptional anti-correlations between the two modules, the correspondence to cellular level proliferation/differentiation switch more unequivocally supports the P/D temporal switch as the switch between proliferation and differentiation.

However, except in the development of compound fly eye [22], it is not known if cellular proliferation/differentiation switches are coordinated at the tissue or individual levels, especially in postmitotic tissues or among post-developmental adult animals. One way the systems level controls are achieved might be through circulating hormones and growth factors, as many of them and their downstream regulation molecules are present at the P–D interface.

### Anti-Correlated Interactions Bridging the P–D Modules

In addition to facilitating the module detection, integrating the interactome and the transcriptome also revealed a large number of PPIs between a limited number of proteins forming a PPI interface between P and D modules. The high degree of interactions at the P–D interface cannot be obtained from randomly generated PPI networks of the same degree distribution as HPRD (compare Control 6 to Experiment 1′ in Table 2; empirical *p* < 0.01, one-tail normal distribution *p* = 1.17 × 10^−8^). The degrees of the P–D interface proteins in sampled PPI networks are also significantly higher than those in the artificially created control networks of the matched degree distributions (compare Controls 7 and 8 with Experiments 4 and 5 in Table 2; one-tail Student *t*-test *p* = 1.66 × 10^−41^ and 1.93 × 10^−63^, respectively).

As expected, anti-correlated interactions preferentially bridge between the transcriptionally anti-correlated P and D modules. More than half (58%) of the correlated interactions are within the coexpressed modules, and 22% are between the P and N modules, whereas 57% of the anti-correlated interactions bridge between the P and D modules, and 22% bridge between the D and N modules (Figure 2C). The probability of the anti-correlated interactions bridging the P and D modules compared with bridging any modules is 1.8 times that of the uncorrelated interactions (PCC between −0.4 and 0.4), which is a very significant difference (Fisher exact test *p* = 2.399 × 10^−20^).

In principle, the anti-correlated interactions can occur among different subjects or in different developmental stages and, as a consequence, bridge various coexpressed modules as small as one- or two-gene modules; therefore, it is surprising to see that two major coexpressed modules comprised 71% of the genes in the network, anti-correlated with each other across 77% of the samples, and were connected by the majority of the anti-correlated interactions in the network.

### Regulatory Role of the P–D Interface

**Figure 4:**
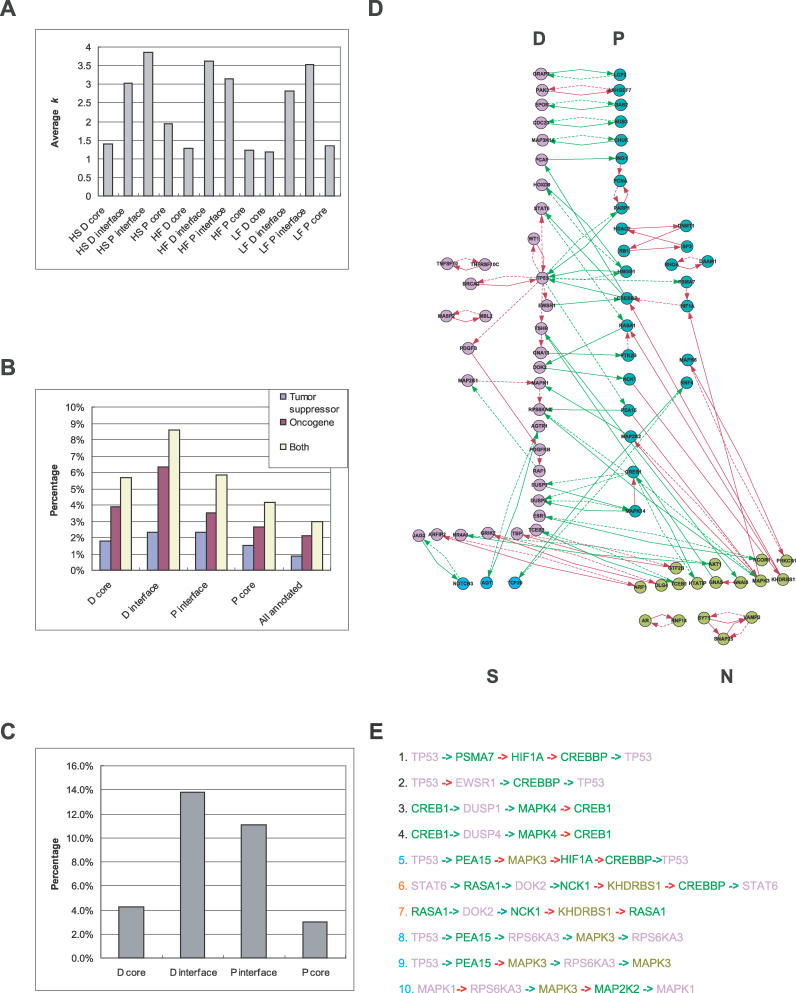
Regulatory Roles of the Protein Interactions between P–D Modules (A) The average degree *k* of the nodes at the human brain and fly P–D protein interaction interfaces and inside the modules (cores). (B) The percentage of proto-oncogenes and tumor suppressor genes at the human brain P–D protein interaction interfaces and inside the modules (cores). (C) The percentage of genes located in feedback loops at the human brain P–D protein interaction interfaces and inside the modules (cores). (D) Network consisting of only the feedback loops traversing protein interaction interfaces and inside the modules (cores). Solid edges represent directional protein interactions; dashed edges, transcriptional regulations. Red and green edges represent transcriptional correlations and anti-correlations, respectively. (E) Feedback pathways potentially controlling P/D switch in the adult human brain. Feedback loops of three or more nodes that traverse the P–D interface are listed. Pathways 5, 8, 9, and 10 are related; 6 and 7 are also related, and they are between all three modules. The font colors for D, P, and N genes are lavender, green, and light green, respectively. Correlated and anti-correlated interactions are shown as red and green arrows, respectively; the arrow points from an upstream to a downstream gene.

From the GO terms overrepresented at the interfaces, the P interface is enriched for transcription factors and the D interface is enriched in cell-cycle checkpoint, DNA repair, and receptor signaling genes (Table 3). All these processes are important regulatory mechanisms in the proliferation/differentiation switch. In particular, the D interface proteins include many of the well-known tumor suppressor genes, such as *BRCA-1* and *p53,* and many receptors and transcription regulators known to be required for neuron differentiation, such as MYC, TOP2B, integrin, estrogen, FGF, PDGF, and TSH receptors, and many A kinase–anchoring proteins (AKAPs), etc. The P interface contains genes promoting cell proliferation, such as *K-RAS, HDACs, SRF, CREB, CREBBP, IL4R,* and *INSR* (the insulin receptor gene). It also contains genes that inhibit p53 and BRCA-1 functions such as *PARC* and *LMO4* (Table 3 and Figure S5). We further evaluated the potential regulatory role of the P–D interface by three well-known network and biological properties of regulatory genes: (1) genes playing crucial regulatory roles are often hubs in the network, and vice versa; (2) if the interface plays crucial regulatory roles in the proliferation/differentiation switch, malfunction of these genes may lead to cancer, and thus these genes are on average more likely to be oncogenes or tumor suppressor genes; and (3) regulatory genes function in regulatory pathways, where feedback control is a dominant network feature. We therefore compared the protein interaction degrees, the percentage of oncogenes and tumor suppressor genes, and the percentage of genes in the feedback loops between the interface and the non-interface, or core genes. The results of all three tests are consistent with a crucial regulatory role in the P/D switch: compared with the core of the P and D modules, the P and D interfaces have a much higher average interaction degree (Figure 4A; *p* = 2.27 × 10^−12^), percentage of known oncogenes and tumor suppressor genes (Figure 4B; *p* = 3.28 × 10^−2^ and 2.08 × 10^−4^ for P and D interfaces, respectively), and percentage of proteins/genes located in feedback loops (Figure 4C; *p* = 1.07 × 10^−2^ and 3.45 × 10^−4^ for P and D interfaces, respectively). Even though all the feedback loops are still of very limited coverage, it is already evident that most of these feedback controls are between the P and D modules and mediated by anti-correlated interactions (Figure 4D). Nearly all the proteins involved in these feedback loops are transcription regulators, and many of the loops are formed by both PPI and transcriptional regulations (Figure 4D). These special features of the P–D interface proteins make them potential key regulators for the proliferation/differentiation switch.

**Table 3:**
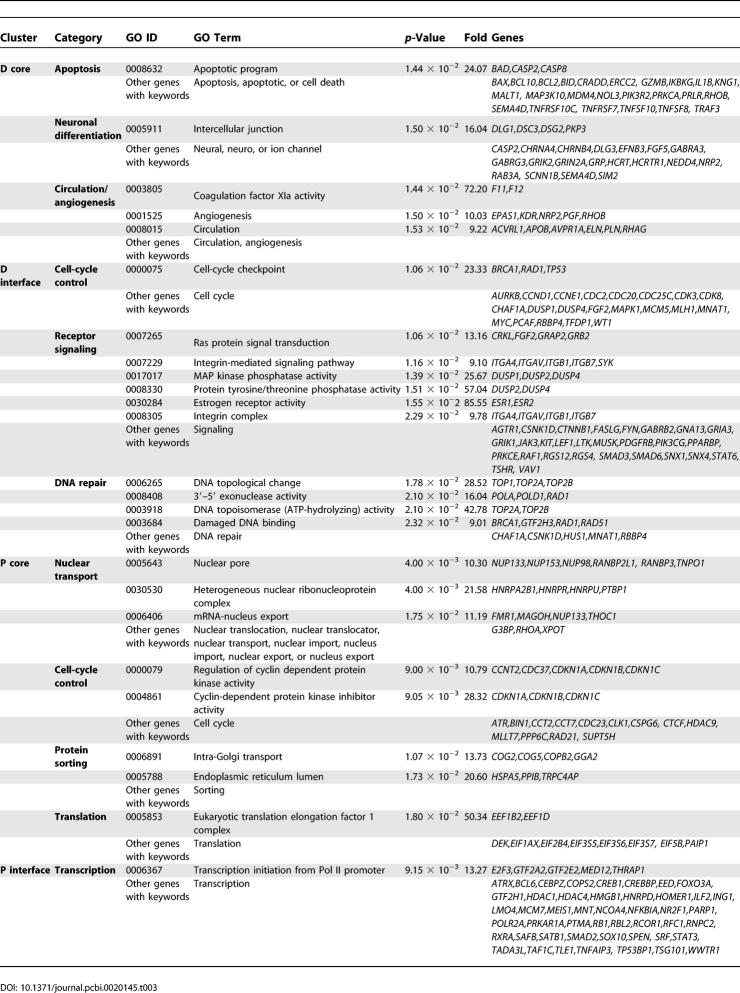
GO Terms Overrepresented in the Interface and Core (Noninterface) of the P and D Modules

Alternating expression of genes can be brought upon by the “toggle switch” network circuit, which is a feedback loop consisting of two mutually inhibitory interactions between the nodes [23]. If we treat the P and D modules as single nodes in a module network, the P and D expression pattern can be also achieved through a simple toggle switch design between them (Xia et al., unpublished data). However, in a complex system involved in differentiation and proliferation control, much more redundancy and fine-tuning than one feedback control might be implemented. As examples of potential feedback controls of the differentiation and proliferation in the adult human brain, we can list at least ten interesting signaling pathways of three or more nodes that traverse the P–D interface (Figure 4E). Highlighting a unique advantage of such integrative systems analysis, these pathways are not just a collection of known pathways preexisting in the literature. Although all the interactions are derived from the literature, and most genes in these pathways have been shown to affect differentiation, proliferation, or growth, the pathways themselves have not been reported previously and are not known as control circuits for coordinating differentiation and proliferation processes.

Altogether, the P and D modules are not only transcriptionally anti-correlated across different individuals, they are also functionally associated with basic cellular proliferation and differentiation functions, evolutionarily represent cell-autonomous and multicellular-specific modules, and are respectively suppressed and induced at the cellular proliferation/differentiation switch in various cells of various multicellular organisms. The functional interdependence, antiphase temporal compartmentalization, and different evolutionary origins of the two modules suggest that the P and D modules are two counterparts in a symbiotic relationship that need to be tightly controlled and coordinated at the cellular, tissue, and organism levels by switching temporally between the two phases—proliferation and differentiation.

## Discussion

In this study, we describe a new integration analysis of the interactome and the transcriptome, which, even though rather straightforward, is very effective at removing the analysis noise of a conventional gene clustering process and allowed us to robustly find the P and D modules and their transcriptional anti-correlation. Moreover, the most important contribution of PPI integration is to reveal the P–D interface, which has a potential regulatory role in coordinating the proliferation and differentiation processes.

We found that anti-correlation goes beyond the individual gene pairs but between the gene populations—a pair of transcriptionally anti-correlated gene groups. The P and D modules seem to be associated with cellular proliferation and differentiation and are suppressed and induced at the cellular proliferation/differentiation switch, respectively, therefore corresponding to alternative states of the cellular network. This indicates that logical relationships also exist at the modular level in the cellular networks. A possible scenario for anti-correlation at the modular level is that it might reflect a temporal separation of the biological functions in the cellular network [24]. The metabolic cycle has been suggested to fulfill such a role of temporal compartmentalization of oxidative and reductive metabolism in eukaryotes [24]. The antiphase temporal compartmentalization of proliferation and differentiation has been demonstrated over and over for single molecules upon switching from proliferation to differentiation at the cellular level [1–4], but it is surprising that such relationships also exist at the tissue or organism level during adulthood or might be brought upon by transcriptionally anti-correlated modules through complex feedback mechanisms between the two modules. Our result is therefore consistent with Waddington's [5] view of the development, in which differentiation and proliferation correspond to two states of the network where a balance between them is achieved at a systems level. More importantly, the tissue-level and organism-level coordination during adulthood and the evolutionary conservation level of the P and D modules imply the balance is not restricted to the single-cell level during early development, but rather exists during the whole life of an organism.

Although such an expression pattern can be achieved by a simple toggle switch between them (Xia et al., unpublished data), in a complex system, redundancy is often implemented to ensure robustness; therefore, multiple toggle switches may exist between the two modules, and the switches must be connected with each other to transfer information. The exact molecular mechanisms giving rise to the transcriptional correlated and anti-correlated modules at the systems level during adulthood remain to be determined; we expect that many signaling pathways involved in cancer formation and aging will be part of the control mechanisms. But due to methodology limitations in the past, most of these pathways have been only studied individually; a general and comprehensive mechanism is still lacking. Our identification of the P and D modules at the systems level has provided an entry point for arriving at such a general mechanism. It is possible that the genes in each module share a few common immediate upstream transcription regulators. For example, we have found that the 5′ untranslated regions of the fly P module genes are clearly enriched for Dref-targeting sites among a few other less-well characterized sequence motifs (Xue et al., unpublished data). Ectopic overexpression of Dref has been shown to block the proliferation/differentiation switch in the fruit fly eye imaginal disc [22].

As the P module is concentrated at transcription-level activities and the N module is concentrated at protein-level activities, a temporal delay between transcription and translation might account for the lack of complete synchronization between the two clusters. Even though the samples are mostly from subjects of different ages, the timescale reflected by these samples may not be restricted to age differences; a delay between translation and transcription may well be reflected as individual differences.

Although the current coverage of the interactome comprising both the literature and large-scale yeast two-hybrid interactions is still limited [25], the conservation of the P–D pattern in the human brain and fruit fly across different datasets indicates that the coverage is sufficient at the current level to detect, annotate, and analyze the P and D modules. In the NP network, we only focused on the strongly correlated and strongly anti-correlated interactions, but the genes excluded this way may also play important regulating functions toward the temporal compartmentalization between P and D modules or in the proliferation/differentiation control. Nevertheless, our identification of the large interconnected P–D modules for the first time revealed a proliferation/differentiation switch and their interrelationship at a systems level. It opens a new avenue to examine differentiation and proliferation at the systems and network levels, and provides a channel to connect physiological level events, such as hormone secretions, to the underlying cellular and molecular changes. It will help to elucidate many complex biological processes.

## Materials and Methods

### Datasets.

The HPRD dataset [14] was downloaded from www.hprd.org on November 22, 2004; a later version of the HPRD dataset was obtained on September 13, 2005; two human Y2H datasets were included in the extended PPI network [19,20]. Two Y2H screens were combined as the fly protein interaction dataset [26,27].

Microarray expression profiles were obtained from previously published studies on postmortem human brains of subjects between 26 and 106 years of age [9] and on adult Drosophila melanogaster of various ages [21].

GO annotations were downloaded from ftp://ftp.ncbi.nlm.nih.gov/gene/DATA on March 10, 2005.

Lists of proto-oncogene and tumor suppressors were obtained at http://ca.expasy.org/cgi-bin/get-entries?KW=Anti-oncogene and http://ca.expasy.org/cgi-bin/get-entries?KW=proto-oncogene on July 4, 2005.

### Orthologs.

Human orthologs in mouse, fly, worm, and yeast were identified as the best reciprocal BlastP hits with an e-value cutoff of 10^−6^ based on RefSeq protein sequences downloaded on December 9, 2004, from http://www.ncbi.nlm.nih.gov/RefSeq.

### Filtering GO terms.

To filter out the GO terms [28] that are broadly associated with many proteins, we calculated the number of proteins each GO term associated with and used only the GO terms that have a detection probability (*p-*value) among randomly paired genes less than the significance value of 0.05 after Bonferroni correction for multiple hypothesis testing on the total number of GO terms in each species. The *p-*value for a GO term is defined as *p* = (n/t)^2^, where *n* is the number of genes associated with the GO and *t* is the total number of genes in the species; a GO term was used only if *p* < (0.05/g), where *g* is the total number of GO terms associated with all the genes in a species.

GO-term enrichment was determined by Fisher exact test followed by Benjamini-Hochberg correction [29] for multiple hypothesis testing on all the GO terms tested in each gene set.

### Feedback loops.

The NP network was searched with the breadth first-search algorithm for pathways that have the same start and end nodes with lengths between two (minimum, one protein and one regulatory edge between two nodes) and ten based on all the directed protein and regulatory interactions we annotated or extracted. Protein interaction annotation was based on the PubMed abstracts of the references listed by HPRD. Regulatory relationships between PPI partners within the NP network were extracted from PubMed by the Pathway Assist text-mining function (Ariadne Genomics, http://www.ariadnegenomics.com/products/pscentral). Other regulatory relationships were predicted from the transcription factor motifs annotated by the TRANSFAC database or from the p53 chromatin immunoprecipitation experiment [30], then filtered with pairwise expression |PCC| > 0.4.

### Expression clustering and visualization.

Unsupervised agglomerative hierarchical clustering of genes was performed in Cluster 3.0 [15,16]. The expression values were first adjusted by the following operations: log transform, median center genes, normalize genes, median center arrays, and normalize arrays. Then, hierarchical uncentered correlation and centroid linkage were used for clustering in both dimensions. The clustering results were visualized in JavaTreeView 1.0.12 [15,17].

The layout of the reorganized NP network to visualize the intercluster interactions was created with a new visualization tool we developed in Java (Hou et al., unpublished data) and imported into Cytoscape 2.1 [31]. All other network visualization was achieved directly with Cytoscape 2.1.

Networks of a predefined degree distribution were generated by an algorithm used by Milo et al. [32].

## Supporting Information

Figure S1 A More Detailed Textual Flowchart of the Pipeline for Revealing Anti-Correlated Modules (240 KB PDF)

Figure S2 The SD and SN Modules and the Interaction Distribution in the SD–SN and DS1–4 Coexpressed Modules (A) SD and SN clusters. The gene expressions of these two clusters are also anti-correlated between two sample clusters across 63% of the samples. The two sample clusters are different from those giving rise to D and P gene differential expressions. The average expression intensities of the genes in the SD and SN modules are also anti-correlated across different individuals with a PCC of −0.744.
(B) Number of correlated and anti-correlated interactions within and between the SD and SN modules.
(C) Number of anti-correlated and correlated interactions within and between DS1 to DS4 modules, and those between the D submodules and the other major modules (P, N, and S modules).
(382 KB PDF)

Figure S3 Overlaps of HPRD Modules to Those Derived from the Extended Minus HPRD PPI Network Even though there is not a single edge or interaction in common between this network and the HPRD network, three modules derived from it share significant overlaps to P, D, and N modules, respectively.
(337 KB PDF)

Figure S4 The P–D Partition Does Not Depend on a PPI Network or PCC Cutoffs, but the Right |PCC| Cutoff Can Facilitate its Identification (A) Hierarchical clustering followed by an automated exhaustive search (for clusters that contain less than 1% of interactions of PCC < 0) identified anti-correlated modules of sizes ranging from 100 to 1,200 genes that significantly overlap the original P and D modules when a more comprehensive PPI network and various PCC cutoffs (including no PCC cutoff) were used to extract NP network. Even without integrating a PPI network, a careful manual search can identify loosely aggregated gene expression clusters of 783 and 1,276 genes that significantly overlap with the P and D modules. When the PCC cutoff is >0.7, no gene cluster that has more than 100 genes can be identified as significantly overlapping with the D module. Input gene sets together with the number of genes in each set (in parentheses) are listed as the row headers on the left. The sizes of the clusters identified under various PCC cutoffs or without a PPI network are listed as the row headers on the right, and those of the original P, D, N, and S modules are listed on the column headers on the top. The number of genes overlapping between the original and latter examined clusters is indicated in each cell of the matrix; the intensity of the background color of a cell reflects the overlap significance as –log (*p-*value) by Fisher exact test normalized by standard deviation among all the cluster overlaps examined, which equals 44. (B) The fractions of P–D genes within the NP network at various |PCC| cutoffs used to extract the NP network from the extended PPI network. A maximal fraction is achieved at |PCC| cutoffs of 0.45 and 0.5 (yellow bars). Because some N module genes are merged into the P modules at |PCC| cutoffs of 0.45 and 0.5, the fractions of the module genes that overlap with the original NP network P and D modules are also plotted to exclude the potential bias introduced by the inclusion of some N genes. The fraction of P and D module genes overlapping with the original P and D modules (blue bars) display a similar trend to the fraction of total genes in each module (yellow bars).
(342 KB PDF)

Figure S5 The P–D Protein Interaction Interface The cores of the modules are represented as big squares on either side of the interfaces.
(239 KB PDF)

Table S1 Gene List of Each Module (161 KB XLS)

Table S2 Sample Information of Cell Differentiation Induction Experiments (137 KB PDF)

Text S1 The P–D Partition Is a Feature of the Expression Pattern in the Cellular Network (11 KB PDF)

## Abbreviations

GO: Gene Ontology
HPRD: Human Protein Reference Database
NP: negative–positive
PCC: Pearson correlation coefficient
P–D: proliferation–differentiation
PPI: protein–protein interaction
