# Supplementary figures and images for: Identification of the Proliferation/Differentiation Switch in the Cellular Network of Multicellular Organisms
Source: PLoS Comput Biol. 2006 Nov 24;2(11):e145. doi: 10.1371/journal.pcbi.0020145 (PMC1664705; doi:10.1371/journal.pcbi.0020145)

## Supplementary Figure 1

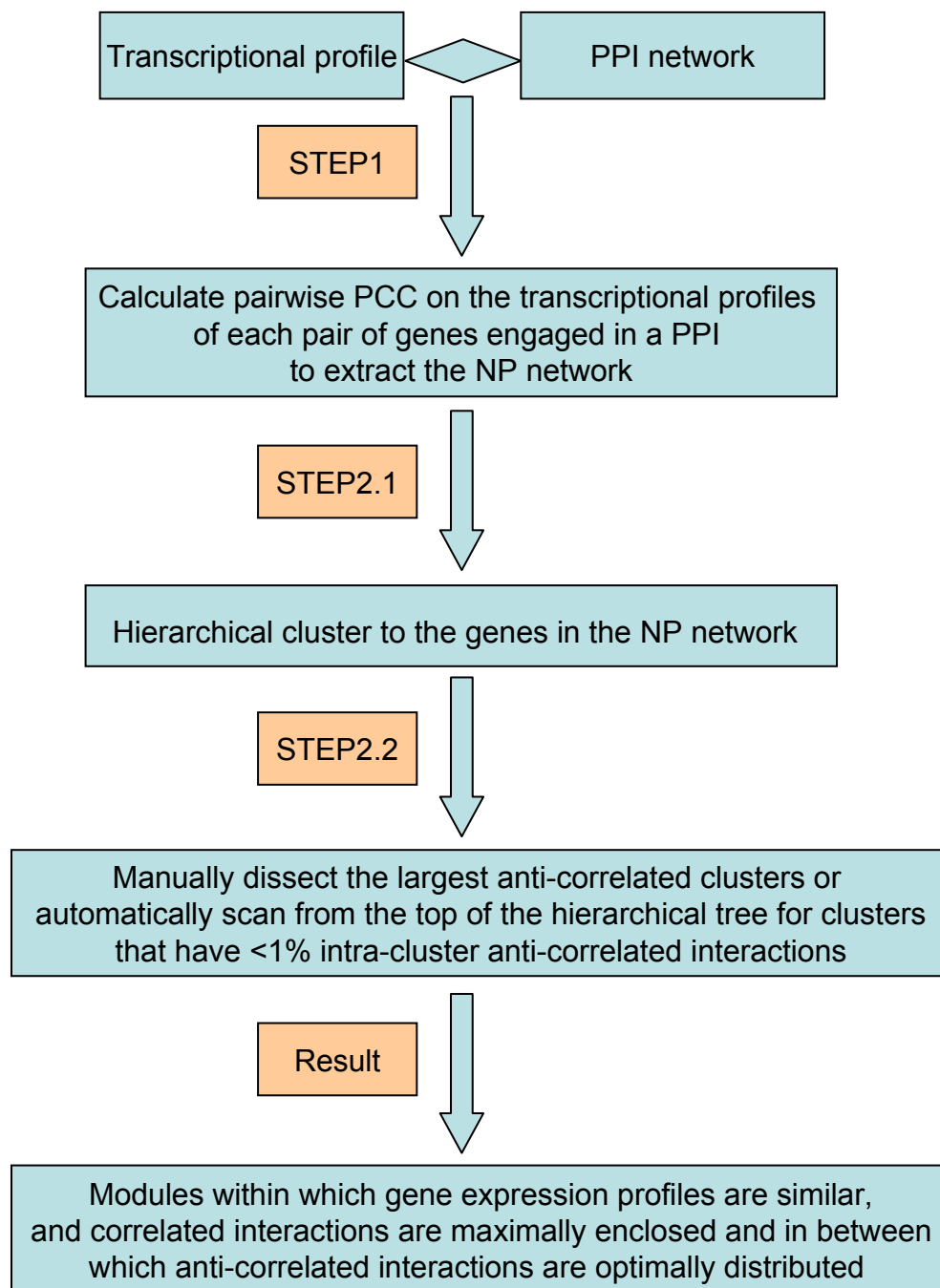

Supplement: Figure S1 — (240 KB PDF) [file pcbi.0020145.sg001.pdf]

# Supplementary Figure 2

A

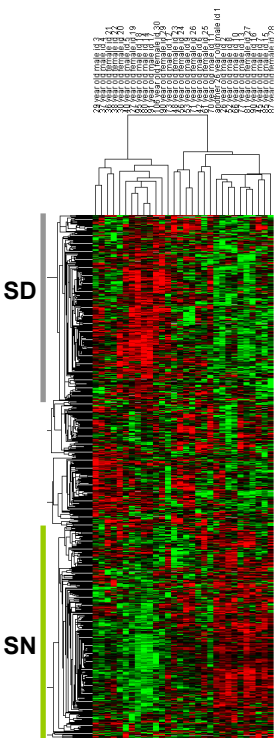

B

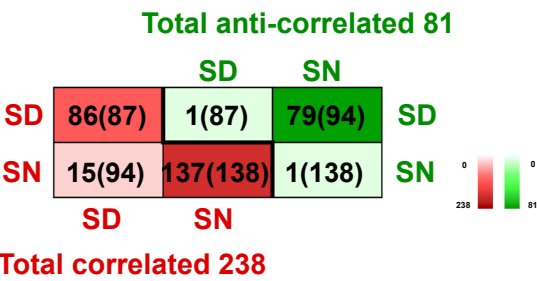

C

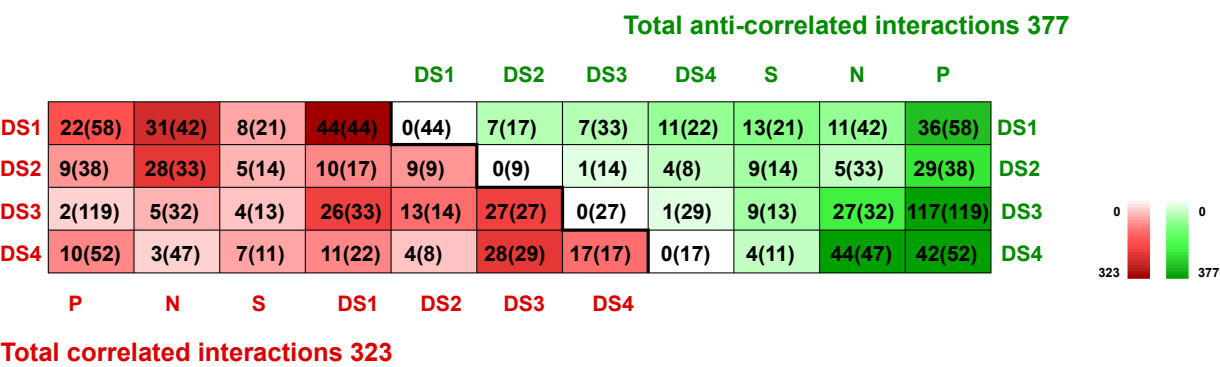

Supplement: Figure S2 — (A) SD and SN clusters. The gene expressions of these two clusters are also anti-correlated between two sample clusters across 63% of the samples. The two sample clusters are different from those giving rise to D and P gene differential expressions. The average expression intensities of the genes in the SD and SN modules are also anti-correlated across different individuals with a PCC of −0.744. (B) Number of correlated and anti-correlated interactions within and between the SD and SN modules. (C) Number of anti-correlated and correlated interactions within and between DS1 to DS4 modules, and those between the D submodules and the other major modules (P, N, and S modules). (382 KB PDF) [file pcbi.0020145.sg002.pdf]

# Supplementary Figure 3

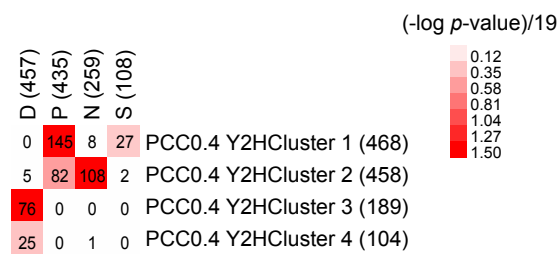

Supplement: Figure S3 — Even though there is not a single edge or interaction in common between this network and the HPRD network, three modules derived from it share significant overlaps to P, D, and N modules, respectively. (337 KB PDF) [file pcbi.0020145.sg003.pdf]

Supplementary Figure 5

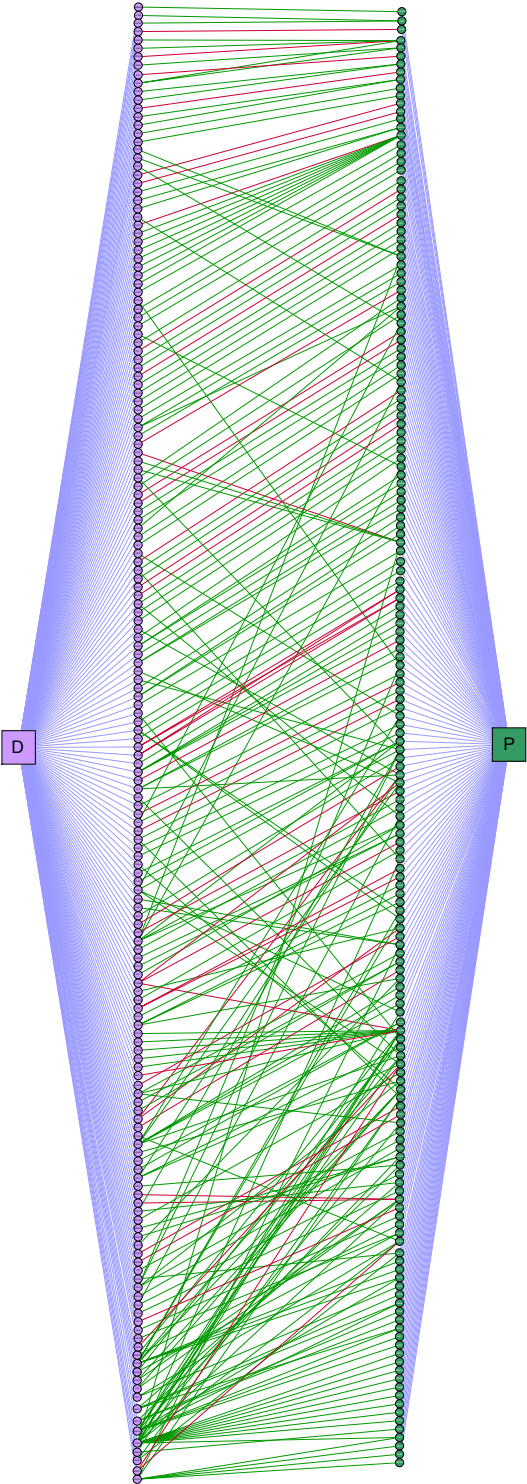

Supplement: Figure S5 — The cores of the modules are represented as big squares on either side of the interfaces. (239 KB PDF) [file pcbi.0020145.sg005.pdf]
