# Supplementary material for: Identification of the Proliferation/Differentiation Switch in the Cellular Network of Multicellular Organisms
Source: PLoS Comput Biol. 2006 Nov 24;2(11):e145. doi: 10.1371/journal.pcbi.0020145 (PMC1664705; doi:10.1371/journal.pcbi.0020145)

Supplementary Figure 4

A

	D (457)	P (435)	N (259)	S (108)	
	226	0	2	2	whole chip Cluster 1 (1276) **
whole chip (7001)	0	194	11	0	whole chip Cluster 2 (783)
	1	232	15	2	PCC0 Cluster 1 (601)
	5	80	139	1	PCC0 Cluster 2 (500)
PCC0 (3973)	113	0	0	1	PCC0 Cluster 3 (339) *
	0	293	11	52	PCC0.3 Cluster 1 (719)
	33	103	192	5	PCC0.3 Cluster 2 (670)
	111	0	0	0	PCC0.3 Cluster 3 (220)
PCC0.3 (2804)	88	0	1	0	PCC0.3 Cluster 4 (214)
	45	62	190	4	PCC0.35 Cluster 1 (607)
	2	256	20	3	PCC0.35 Cluster 2 (508)
	138	0	0	0	PCC0.35 Cluster 3 (252)
PCC0.35 (2528)	0	80	1	30	PCC0.35 Cluster 4 (241)
	4	282	19	65	PCC0.4 Cluster 1 (656)
	14	128	198	6	PCC0.4 Cluster 2 (603)
PCC0.4 (2186)	216	0	1	1	PCC0.4 Cluster 3 (347)
	18	399	188	82	PCC0.45 Cluster 1 (1176)
PCC0.45 (1840)	148	0	0	1	PCC0.45 Cluster 2 (228)
	9	357	173	52	PCC0.5 Cluster 1 (971)
PCC0.5 (1502)	130	0	1	1	PCC0.5 Cluster 2 (203)
	3	209	20	35	PCC0.6 Cluster 1 (419)
	16	72	110	1	PCC0.6 Cluster 2 (323)
PCC0.6 (939)	96	0	0	2	PCC0.6 Cluster 3 (133)
PCC0.7 (479)	1	119	11	15	PCC0.7 Cluster 1 (227)

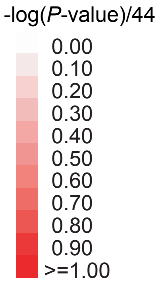

* This cluster is merged by two smaller clusters because the number of cluster is less than 100.

** These two clusters were manually extracted from the clustering dendrigraph of the whole gene in U95 genechip based on visual anti-correlation.

B

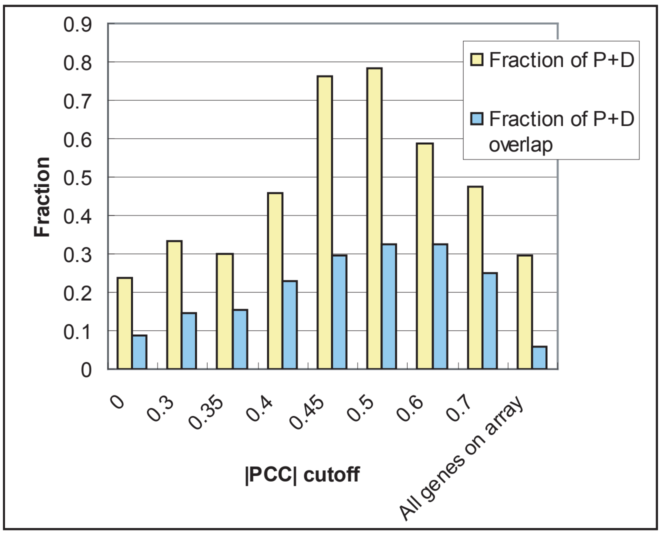

Supplement: Figure S4 — (A) Hierarchical clustering followed by an automated exhaustive search (for clusters that contain less than 1% of interactions of PCC < 0) identified anti-correlated modules of sizes ranging from 100 to 1,200 genes that significantly overlap the original P and D modules when a more comprehensive PPI network and various PCC cutoffs (including no PCC cutoff) were used to extract NP network. Even without integrating a PPI network, a careful manual search can identify loosely aggregated gene expression clusters of 783 and 1,276 genes that significantly overlap with the P and D modules. When the PCC cutoff is >0.7, no gene cluster that has more than 100 genes can be identified as significantly overlapping with the D module. Input gene sets together with the number of genes in each set (in parentheses) are listed as the row headers on the left. The sizes of the clusters identified under various PCC cutoffs or without a PPI network are listed as the row headers on the right, and those of the original P, D, N, and S modules are listed on the column headers on the top. The number of genes overlapping between the original and latter examined clusters is indicated in each cell of the matrix; the intensity of the background color of a cell reflects the overlap significance as –log (p-value) by Fisher exact test normalized by standard deviation among all the cluster overlaps examined, which equals 44. (B) The fractions of P–D genes within the NP network at various |PCC| cutoffs used to extract the NP network from the extended PPI network. A maximal fraction is achieved at |PCC| cutoffs of 0.45 and 0.5 (yellow bars). Because some N module genes are merged into the P modules at |PCC| cutoffs of 0.45 and 0.5, the fractions of the module genes that overlap with the original NP network P and D modules are also plotted to exclude the potential bias introduced by the inclusion of some N genes. The fraction of P and D module genes overlapping with the original P and D modules (blue bars [file pcbi.0020145.sg004.pdf]
