# Supplementary material for: Identification of the Proliferation/Differentiation Switch in the Cellular Network of Multicellular Organisms
Source: PLoS Comput Biol. 2006 Nov 24;2(11):e145. doi: 10.1371/journal.pcbi.0020145 (PMC1664705; doi:10.1371/journal.pcbi.0020145)
Supplement: Table S2 — (137 KB PDF) [file pcbi.0020145.st002.pdf]

Supplementary Table 2

Microarray dataset	Negative control	Positive control	Reference
HS endometrial stromal cell differentiation by cAMP	0-2 hr	12 hr	PMID: 14532334
MM myoblast differentiation	-2 to -1 day	2 to 10 day	PMID: 14688207
MM smooth muscle cell differentiation by RA	untreated	RA treated	PMID: 15340120
RN chondrocyte differentiation by FGF1	0 hr	1-24 hr	PMID: 12821644
DM neural progenitor cell differentiation	cholinergic/Gad1 negative	neural differentiation positive	GEO ID: GSE1060
