# Supplementary material for: Identification of the Proliferation/Differentiation Switch in the Cellular Network of Multicellular Organisms
Source: PLoS Comput Biol. 2006 Nov 24;2(11):e145. doi: 10.1371/journal.pcbi.0020145 (PMC1664705; doi:10.1371/journal.pcbi.0020145)
Supplement: Text S1 — (11 KB PDF) [file pcbi.0020145.sd001.pdf]

Text S1. P-D partition is a feature of expression pattern in the cellular network

In addition to |PCC|>0.4, we also tested other PCC cut-offs (|PCC| > 0, 0.3, 0.35, 0.4, 0.45, 0.5, 0.6 or 0.7) to extract the NP network. The clustering method is applied as discussed in the main text and an automated program is used to divide the NP network genes into clusters. The program takes the cluster results from the Cluster program as input, scan through clusters at increasing level from the root dendrite of the clustering tree. If a cluster has fewer than 1% intra-cluster interactions of PCC<0, then the cluster is selected as a final cluster and all the genes within the cluster are removed from further scanning, until all levels of the tree are scanned. We then examined the cluster profiles, the intra- and inter-cluster protein interactions and the cluster gene overlaps to those of the |PCC|>0.4 NP network. Under all circumstances, the P, D and N clusters are all present in the clustering profile although by the automated cluster dividing method, the D is sometimes divided into 2 or 3 smaller sub-clusters (DSs), and the N is merged into the P when the cut-off is 0.45 or 0.5. P usually has the largest number of genes. Even when the cut-off is set to 0.7, we still found a cluster of 160 genes, out of which 136 overlap with the original P module of the |PCC|>0.4 NP network, but D module genes start to disappear at |PCC|>0.7, which is consistent with the signaling related functions and the pathway-like structures of the D module (Figure S4A). By our modular interaction layout graph, we were always able to see the modular interactions among P, D and N. While the PCC cut-off ascends, the P-D modules became more and more enriched in the clustering tree view and the P-D partition became clearer and clearer on the modular network layout graph, although with fewer and fewer nodes.

However, the |PCC| cutoffs used to extract the NP network may serve to optimize the P-D module identification, as shown in Supplementary Figure 4B, the fractions of P-D modules relative to the genes in the NP network or the whole number of genes before expression profile clustering increase as |PCC| cutoffs increase from 0 to 0.45, decrease after 0.5. In other words, at |PCC|

cutoff of 0.45 or 0.5, the P-D modules can be maximally identified. The effect of |PCC| cutoff on the easiness of P-D modules identification is also seen in 4 and 5 of Table 2, when NP network extraction was omitted from the analysis process, only 60-68% of the time, we can identify anti-correlated modules, with 166 nodes each module on average, as compared to nearly 100% of the times and an average 489 nodes per module when |PCC| cutoffs between 0.3 and 0.7 are used.
